# Radial Head Prosthesis with Interconnected Porosity Showing Low Bone Resorption Around the Stem

**DOI:** 10.3390/jcm14155439

**Published:** 2025-08-01

**Authors:** Valeria Vismara, Enrico Guerra, Riccardo Accetta, Carlo Cardile, Emanuele Boero, Alberto Aliprandi, Marco Porta, Carlo Zaolino, Alessandro Marinelli, Carlo Cazzaniga, Paolo Arrigoni

**Affiliations:** 1 Scuola di Specializzazione in Ortopedia e Traumatologia, Università Degli Studi Di Milano, 20122 Milan, Italy; vale.vismara@gmail.com; 2 Shoulder and Elbow Unit, IRCCS Istituto Ortopedico Rizzoli, 40136 Bologna, Italy; enrico.guerra@ior.it (E.G.); alessandro.marinelli@ior.it (A.M.); 3 IRCCS Orthopaedic Institute Galeazzi, 20161 Milan, Italy; 4 ASST-Rhodense Ospedale di Garbagnate Milanese, 20024 Garbagnate Milanese, Italyccazzaniga@asst-rhodense.it (C.C.); 5 Orthopaedic and Trauma Unit, Ospedale Camposampiero (Padova), AULSS 6 Euganea, 35012 Camposampiero, Italy; emboero@gmail.com; 6 Radiology Unit, Zucchi Clinical Institutes Spa, 20900, Monza, Italy; alberto.aliprandi@grupposandonato.it (A.A.); marco.porta@grupposandonato.it (M.P.); 7 Clinica Ortopedica, Azienda Socio Sanitaria Territoriale Centro Specialistico Ortopedico Traumatologico Gaetano Pini-CTO, 20122 Milan, Italy; carlo.zaolino@gmail.com

**Keywords:** radial head replacement, radial head trauma, radiological outcomes, bone resorption, 3D-printed radial head prosthesis

## Abstract

**Background/Objectives**: Radial head arthroplasty is a commonly preferred treatment for complex, unreconstructable radial head fractures. Recent studies have raised the question of whether factors such as bone resorption may be related to failure. This observational, retrospective, multicenter, spontaneous, and non-profit study aims to assess radiological outcomes, focusing on bone resorption around the stem, for radial head replacement using a modular, cementless radial head prosthesis with interconnected porosity. **Methods**: A series of 42 cases was available for review. Patients underwent radial head arthroplasty using a three-dimensional-printed radial head prosthesis. Patients were eligible for inclusion if they had undergone at least one follow-up between 6 and 15 months post-operatively. A scoring system to detect bone resorption was developed and administered by two independent evaluators. **Results**: Forty-two patients (14 males, 28 females), with an average age of 59 ± 11 years (range: 39–80 years), were analyzed with a minimum of six months’ and a maximum of 32 months’ follow-up. At follow-up, 50 radiological evaluations were collected, with 29 showing ≤3 mm and 12 showing 3–6 mm resorption around the stem. The average resorption was 3.5 mm ± 2.3. No correlation was found between the extent of resorption and the time of follow-up. The developed scoring system allowed for a high level of correlation between the evaluators’ measurements of bone resorption. **Conclusions**: Radial head prosthesis with interconnected porosity provided a low stem resorption rate for patients after a radial head fracture at short-to-mid-term follow-up after the definition of a reliable and easy-to-use radiological-based classification approach. (Level of Evidence: Level IV).

## 1. Introduction

Radial head fractures account for up to 4% of all adult fractures and roughly 33% of elbow fractures [1]. This type of fracture occurs after a fall on the outstretched hand, with the forearm pronated and the wrist extended [2]. Concomitant lesions are present in nearly 80% of multi-fragment fractures [3]. Depending on the fracture type, management may be conservative or surgical. Based on the Mason classification [4], three types of fractures may occur: Type I, which is a fissure or marginal fracture without displacement; Type II, which is a displaced marginal fracture with separation or impaction; and Type III, which is a displaced comminuted fracture involving the whole head of the radius. Management is non-operative for Type I fractures, while reduction and internal fixation are required for Type II [5]. When anatomic reduction and stable internal fixation, allowing for early mobilization, are too difficult or impossible to achieve, as is the case in Type III fractures, radial head replacement (RHR) is a valid alternative.

Radial head replacement serves as an alternative to the technically demanding internal fixation or excision procedures. In previous years, a wide array of radial head prostheses have been introduced to the market based on several differences regarding their geometry, modularity, materials, or fixation techniques [6]. For RHR, many implants are cementless stems with either press-fit ingrowth or a smooth, loose-fitting stem [6]. Despite their wide use, implant-related problems may create situations that are challenging to manage. Typical indications for the removal or revision of radial head arthroplasty are prosthesis loosening and malpositioning, including overstuffing, which may lead to pain. Radial head loosening has been associated with, among other consequences, cortical expansion of the radial neck and bone loss [7]. Thus far, an available comprehensive classification method for a cut-off value for an acceptable degree of resorption around the radial neck is lacking. Few reports [8,9] have suggested a length of ≤3 mm as a good outcome, as this minor and non-progressive resorption would not likely compromise implant fixation [10]. Overall, these issues raise the question of whether factors related to implantation or fixation may be related to early failure.

Except in the case of silicone prostheses, which have previously been shown to be insufficient from a biological and biomechanical point of view, with a substantial risk of implant fragmentation [11,12], it is not clear which type of metal prosthesis is superior, despite several studies focusing on the evaluation of the functional and radiographic outcomes of various prosthetic models. A recent technological push in prosthetic tribology, represented by three-dimensional (3D)-printed titanium, has allowed the creation of a new model of prosthetic stem with possibly greater holding and osteointegration capabilities compared with previous ones [13,14].

This study aimed to evaluate and measure the resorption of a cementless radial head implant with interconnected porosity at short-to-mid-term follow-up using 3 mm as a benchmark for positive outcome, based on available data from the literature [8,9,10]. The secondary objective was to evaluate the inter-observer variability of the radiological measurements taken with an easy-to-handle radiograph-based evaluation system, in order to confirm the reliability of the classification system.

## 2. Materials and Methods

### 2.1. Study Design

The study was conducted in accordance with the Declaration of Helsinki and was approved by the Ethics Committee Milano Area 2 (protocol code EBI-RHR, approved on 18 February 2021). Informed consent was obtained from all subjects involved in the study. The study was observational, retrospective, multicenter, spontaneous, and non-profit. Medical history data were collected on the day of surgery, while radiological evaluations were performed both post-operatively on the same day and during follow-up visits; the patients included were required to have had at least one follow-up between 6 and 15 months. The mean analyzed period for all included follow-ups was 9 ± 5 months (range, 6–32 months).

### 2.2. Patient Selection

This study aimed to recruit functional, pain-free patients who had undergone prosthetic replacement of the radial head and to analyze the radiographs collected during routine post-operative follow-up checks. The inclusion criteria were as follows: (i) a patient age at the time of surgery of 18–80 years; (ii) patients who received, understood, and signed the informed consent form for participation in the study; (iii) patients who were able to understand the conditions of the study and participate throughout its duration; (iv) a radial head fracture (Mason II or III) that could not undergo fixation due to either poor bone quality or the multi-fragmented nature of the fracture; (v) the same cementless radial head implant with interconnected porosity was used for RHR; and (vi) a new implant was included when the prosthesis was replaced with a prosthesis of the same family, and only when not related to first implant failure. We excluded patients for whom radiographic or clinical documentation corresponding to the post-operative period could not be found. After applying the inclusion and exclusion criteria, 42 patients were included in the study.

### 2.3. Prosthesis Features

All patients in the cohort received the same radial head implant (ANTEA radial head implant, Adler Ortho SpA, Cormano, Italy) composed of three elements: (i) an X-ray transparent radial head made of ultra-high-molecular-weight polyethylene and covered with a TiNbN-coated titanium alloy cap; (ii) an intermediate coupling element with a spherical head, allowing for the self-alignment of the radial head, made of TiNbN-coated titanium alloy; and (iii) a cementless 3D-printed radial stem. This element has a monolithic ingrowth surface with interconnected 3D porosity.

### 2.4. Data Collection and Storage

An alphanumeric code uniquely identified each participant. All the data required by the protocol were recorded by an investigator who directly followed the study and its correct conduct for its entire duration from May 2018 to July 2022. The radiological data were collected and stored in digital form, while all remaining data were initially recorded on paper forms. A file containing all the completed forms was kept for each patient. During the study, the data were progressively entered into an electronic database, and a constant backup was made. This study is reported in accordance with the STROBE checklist for observational studies.

### 2.5. Radiological Evaluation

The radiographs taken routinely during the post-operative period and during the clinical follow-up visits were evaluated starting from 6 months post-surgery to limit exposure to ionizing radiation. For all patients, serial plain radiographs, including antero-posterior (AP) and lateral (LL) views, were used to evaluate bone resorption by two blinded and independent reviewers who were not involved in patient treatment. All radiographs were evaluated using a standardized anatomical reference point, namely the bone–implant interface at the stem collar, to localize the measurement site. This enabled consistent identification of the region of interest (ROI), despite variations in patient positioning. All X-rays were taken with the forearm in neutral rotation and with a radiographical landmark so that measurements could be reproducible among the different centers.

### 2.6. Evaluation of Resorption

**Figure 1:**
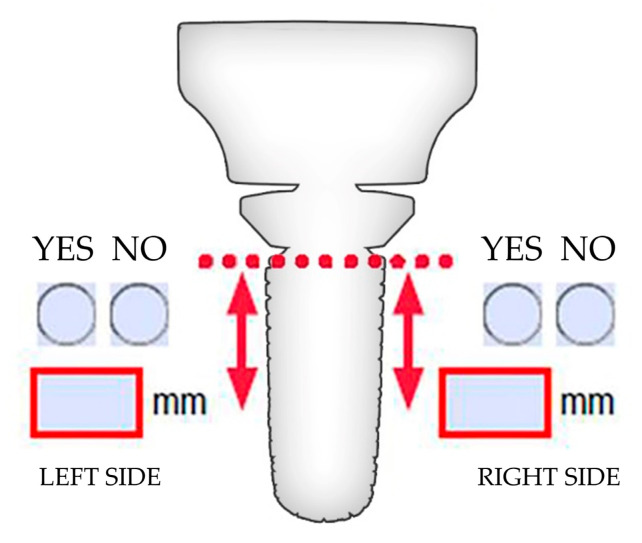
Evaluation of bone resorption. An example of the scheme provided to the independent evaluators to quantify the presence or absence of bone resorption (YES or NO) on the right and left sides of the stem and, in the case of a positive answer, the length of bone reduction. The same scheme was used for the antero-posterior (AP) and lateral (LL) views.

To obtain a reliable estimation of bone resorption, we developed a radiograph-based system (Figure 1) following a simple scheme that could also be applied to other implants with similar features. Bone resorption was evaluated for the left and right sides of the stem in the AP and LL views, referring to how it was positioned in the immediate post-operative radiograph. If the stem was positioned 1 mm or 2 mm more proximally, then the resorption was measured accordingly. Moreover, resorption was measured concerning the stem, not starting from the lower part of the intermediate module, considering that the intermediate module is always a few millimeters outside the bone. This system was used by two expert radiologists who were blinded to, independent of, and not involved in the patients’ treatment.

### 2.7. Data Analysis

The data are tabulated and described as frequencies and cumulative frequencies. The median ranks are also presented. Correlation analyses were performed using Pearson correlation coefficients. A comparison between values was performed with a mixed-effects model using paired values, while a comparison of resorption with a benchmark, with 3 mm as the hypothetical value, was performed using a sample t-test. Statistical significance was set to *p*-value ≤ 0.05. Statistical analyses were conducted using GraphPad Prism software v8 (GraphPad, San Diego, CA, USA).

## 3. Results

The mean age of the patients was 59 ± 11 years (range, 39–80 years). One patient had bilateral surgery (cases 39/40). Revision surgery was carried out on only one patient (case 4) following a prosthesis-unrelated post-traumatic break of the radius, which occurred after the 6-month follow-up, and was thereby included in the initial series of cases. The same patient received a replacement from the same implant family (case 5) and underwent a subsequent clinical and radiographic follow-up at 10 months, and was also included in our final series. No patients underwent revision for loosening of the implant. A total of 44 implants were analyzed across 42 patients (Table 1).

For the only revision case after a stem break (case 5 vs. case 4), a longer spacer (17 mm vs. 13 mm for a final implant collar offset of 5 mm vs. 1 mm) and a smaller radial head (17.5 mm vs. 19 mm) were used. Overall, a wide array of combinations were used, with the most preferred one being a stem size of 9/19.7 mm (diameter/length), with a spacer/collar onset of 13/1 mm and a radial head diameter of 19 mm (four cases). The other most-used combinations (three cases each) were a stem size of 8/19.4 mm (stem diameter/length) with a spacer/collar offset of 13/1 mm and a head diameter of 19 mm, and a stem size of 10/20 mm with a spacer/collar offset of 13/1 mm and a head diameter of 20.5 mm. Heterogeneity in the assembly of prosthesis components was also scored using Pearson correlation analyses of the five values: stem diameter/length, spacer/collar lengths, and head diameter. After data cleaning for the “*r*” values of 1 given by coupled parameters, such as stem diameter/length or spacer/collar length, a low correlation appeared for all datasets, with a stem versus collar of −0.131 (*p*-value > 0.05), collar versus head of 0.214 (>0.05), and head versus stem of 0.430 (0.004). The significant *p*-value for the latter with the highest *r* value suggests a positive correlation between stem and head size (the largest stem preferred with the largest head).

**Figure 2:**
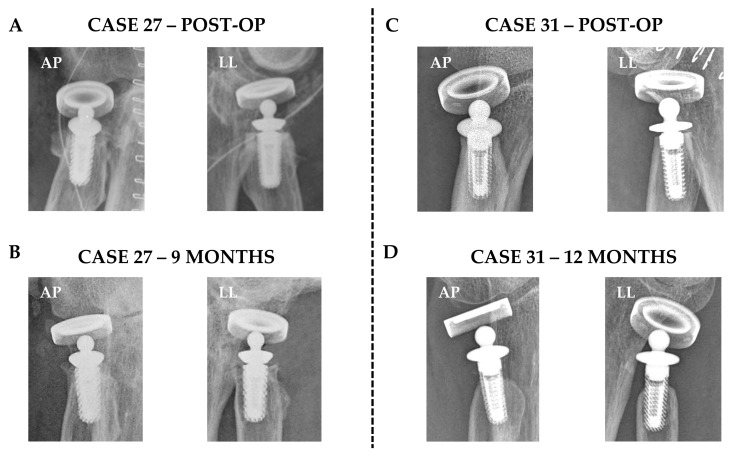
Radiographs of patients without and with bone resorption. (**A**,**B**) Antero-posterior (AP) and lateral (LL) radiographs of case 27 showing no resorption at 9 months’ follow-up compared with post-op. In each radiograph, the embedded ruler was used to calculate resorption in mm. (**C**,**D**) AP and LL radiographs of case 31 showing resorption at 12 months’ follow-up compared with post-op.

For resorption detection (Figure 1 for the new scheme developed in the study and Figure 2 for two cases without and with bone resorption), the Pearson correlation between the evaluators was measured for all scored parameters, namely the left and right sides of the stem, in the AP or LL view. The results showed an *r* of 0.744 (*p*-value < 0.001) for the left AP, 0.791 (*p*-value < 0.001) for the right AP, 0.749 (*p*-value < 0.001) for the left LL, and 0.721 (*p*-value < 0.001) for the right LL. These results confirmed the high consistency between evaluators; thus, the merged values were calculated (Table 2, Panel A). Pearson correlation was calculated for these values, and high *r* values (<0.6) with significant *p*-values (≤0.05) emerged (Table 3). This confirmed similar resorption, if present, on all sides of the stem; therefore, a mean value for each case was provided (Table 2, Panel B). Furthermore, correlation analyses between this value and the age of the patients or the follow-up months after surgery were conducted. Notably, no correlation was found for the tested parameters, with an *r* of −0.164 for resorption versus age (*p*-value of 0.25) and 0.072 for resorption versus month of follow-up (0.62). Eventually, for the population of the study, the mean resorption was calculated to be 3.5 mm ± 2.3, with a median of 2.9 (min–max 0–9) and 29 evaluations (58%) ≤ 3 mm. When comparing the mean value of the series with the cut-off of 3 mm as the benchmark for reduced resorption and positive outcomes, no statistical significance emerged (*p*-value of 0.13). Again, the homogeneity of resorption around the stem was confirmed using a paired mixed-effects model analysis, which showed no statistical differences between the single groups used to calculate the mean resorption value. The left AP resorption was 3.3 mm ± 2.8, the right AP resorption was 3.5 ± 2.6, the left LL reportion was 3.7 ± 2.4, and the right LL resorption was 3.2 ± 2.4; for the same categories, the median values were 3 mm (min–max 0–11), 3 mm (0–9), 4 mm (0–9), and 3 mm (0–9), respectively.

## 4. Discussion

The main finding of this study is that with our established method for bone resorption, the majority of cases (58%) showed reduced bone resorption around the radial head implant, with a mean of 3.5 mm ± 2.3 (median of 2.9 mm), and no statistically significant difference was observed compared to the 3 mm benchmark defined as the threshold for a favorable outcome, at short-to-mid-term follow-up (50 f.u., 6–32 months). The newly developed radiological system allowed for straightforward and consistent assessment of stem resorption, and its efficacy was proven by two blinded and independent evaluators of radiographic images. This scheme may be applied to other implants with similar features.

Radial head arthroplasty is predominantly used for the treatment of acute multi-fragmentary radial head fractures or their sequelae, such as pseudoarthrosis, post-traumatic arthritis, and instability [15,16]. Over the past 75 years, moderate-to-good outcomes have been reported for both primary [6,17] and revision surgery, with rates for the latter reported to be as high as 8% over four years [6]. Recent studies have shown conflicting 10-year survival rates of 61% and 97% [17,18]. This raises the question of whether factors related to implantation or fixation could be related to early failure. Since the introduction of the radial head prosthesis in 1941, numerous design and material variations have been proposed. Accordingly, modular models, bipolar models, and different fixation methods have been designed [19]. In terms of fixation, cementless radial head prostheses have lower reoperation and complication rates than cemented prostheses [20]. Concerning materials, except in the case of silicone prostheses, which have previously been shown to be insufficient from a biological and biomechanical point of view, with a substantial risk of implant fragmentation [11,12,21], it is not clear which type of metal prosthesis is superior, despite several studies focusing on the evaluation of the functional and radiographic outcomes of the various prosthetic models [22]. Therefore, in the absence of a broad consensus, the novelty of this study lies in its provision of a simple classification system for evaluating bone resorption and for the easy generation of reliable data on resorption between independent evaluators, which can be used and compared in future studies. The proposed radiological system proved to be easily reproducible, reliable, and easy to interpret.

A recent technological push in prosthetic tribology, represented by 3D-printed titanium, has allowed the creation of a new model of prosthetic stem with possibly greater holding and osteointegration capabilities compared with previous ones [13,14]. The interaction between the prosthesis and the bone is the foundation of the long-term success of 3D-printed titanium implants used to rehabilitate patients in orthopedics and dental/maxillofacial surgery [23]. Among the factors considered to be crucial for achieving stability and effective osteointegration, the surface of titanium implants is considered decisive. Adequate roughness is a fundamental characteristic of the osteointegrative process and favors the activities of osteogenic cells and the absorption of plasma proteins that regulate osteoblastic adhesion [24,25]. Surface treatment and manufacturing techniques may also have a great effect, as the ideal surface for better mechanical stability and osteointegration capacity is the one with the highest purity of titanium and adequate roughness [23]. The implant subject of this study was produced using 3D printing manufacturing technology. The hypothesis is that such a technology used for radial stem cementless fixation could somehow provide an advantage in terms of implant primary stability and integration with the hosting bone due to interconnected 3D porosity [26] (average pore size: 700 μm, surface porosity: 65%). Notably, as a crucial factor in defining roughness, pore size affects the osteointegration of the implant [27,28]. A study on rabbits comparing titanium implants with three different pore sizes (500, 700, and 900 μm) showed that the best bone–titanium interfacial strength was achieved when the pore size was 700 μm [29], a value comparable with the pores of the prosthesis tested in this study. Similar results were obtained in an in vivo study on rabbits in which 600 μm pore-sized titanium implants showed the most favorable fixation and osseointegration [30].

Our series, which is one of the largest evaluating RH stem bone resorption, showed good results at short-to-mid-term follow-up (mean follow-up time of 9 ± 5 months; range: 6–32 months), with one case of revision due to a prosthesis-unrelated post-traumatic break of the stem. The reduced average resorption around the stem for the 44 analyzed implants was 3.5 mm (median 2.9). Out of 50 analyzed follow-ups, resorption was ≤3 mm in 29 follow-ups, 3–6 mm in 12 follow-ups, and >6 mm in 9 follow-ups. Considering the series described in this study, no statistical significance was found overall concerning the value of 3 mm which was proposed as the benchmark for a positive implant outcome in the first published classification study [8], and was later supported by other reports [9,10]. Moreover, considering this pioneering classification study, a higher percentage of low-resorption (≤3 mm) patients was observed in our series (58% vs. 33%), in contrast with the trend of intermediate cases (3–6 mm, 24% vs. 54%). The poor outcome rates were similar (18% vs. 13%). Moreover, no correlation was observed between the extent of bone resorption and the follow-up time, suggesting the absence of major resorption over time. These data were further supported by the limited increase in bone resorption for the four patients with a series of follow-ups (cases 2, 31, 32, and 33). Cases 2 and 31 exhibited almost absent and detectable resorption, respectively, remaining identical over time, while for cases 32 and 33, their very-limited-to-moderate resorption increased only by 1 mm over 1.5 years. These results suggest that for the implants reported in this study, mild bone resorption could occur at initial times after surgery and remain constant over time. This is in agreement with previous findings showing that radiolucencies around a loose-fitting stem remain stable after two years [31], although these data were reported for a monopolar implant. Due to the limited available data on longer follow-ups in our series, these suggestions should be further evaluated and studied. Moreover, future studies should be conducted on the choice of the most effective type of prosthesis over time, as other studies have reported progressive resorption of the capitellum after insertion for metal [32], pyrocarbon [33], and porous [34] coated titanium stem radial head prostheses.

Future directions arising from this work are relevant to both clinical practice and research. For clinicians, the proposed radiological classification offers a simple, reproducible tool for monitoring bone resorption around radial head prostheses in routine follow-up. It may assist in identifying abnormal resorption patterns early, guiding decisions on closer monitoring or potential revision. For researchers, this method provides a standardized approach to quantifying resorption and enables reliable comparisons across implant types and studies. Prospective investigations should aim to correlate radiological findings with functional and clinical outcomes, such as pain, mobility, and revision rates. In addition, future studies should explore the influence of prosthesis design, surface topography, and fixation technology, particularly the impact of 3D-printed porous titanium, on osseointegration and long-term implant survival. Studies extending beyond the mid-term period are especially warranted to confirm the long-term stability suggested by our findings.

The authors are aware that this study has some limitations. Its main drawback is its retrospective and descriptive nature. The choice to include patients with follow-up of 6–15 months was both a strength of the study, allowing for a focus on short-to-mid-term outcomes, and a limitation, due to the exclusion of patients with a tendency to skip early intervention and return to clinicians only when problems occur several months after surgery. X-ray evaluation of all patients at longer follow-up is needed to confirm the absence of progressive resorption in the long term, as in this series, only four cases were evaluated within 15 months and later re-evaluated. Moreover, various surgeons and centers with different experiences participated in this study, which could constitute another limitation of the results obtained. To reduce this bias, especially for X-ray data interpretation, independent evaluators were included. On the other hand, the consistency of the results and the overall low bone resorption rate (58%), regardless of the surgeon’s experience and the working center, are promising. In addition, while our study focused specifically on additively manufactured radial head (RH) prostheses, direct comparison with traditionally manufactured implants (e.g., forged or machined metallic stems) was limited by the lack of head-to-head trials and the heterogeneity of existing studies in terms of implant design, material, fixation method, and follow-up duration.

## 5. Conclusions

A low (≤3 mm) bone resorption rate for an RHR prosthesis with a cementless 3D-printed stem produced using additive manufacturing technology was reported in this study. The developed procedure for resorption quantification was demonstrated to be feasible and reproducible by independent evaluators. At short-to-mid-term follow-up, a low rate of revision occurred, with no events related to resorption and loosening.

## Figures and Tables

**Table 1:** Details of the 42 patients in this series treated with radial head replacement.

Patient			Implant Prosthesis			
Case	Gender	Age	Radial Stem Size Ø/L (mm)	Spacer/Collar Offset L (mm)	Radial Head Ø (mm)	Follow-Up Month
1	F	48	8/19.4	13/1	19	8
2	M	56	12/20.9	13/1	20.5	6/12
3	F	62	9/19.7	13/1	19	9
4	F	48	7/19.1	13/1	19	6
5	F	48	7/19.1	17/5	17.5	10
6	F	80	9/19.7	13/1	20.5	6
7	M	47	10/20	13/1	22	15
8	F	49	8/19.4	14.5/2.5	19	7
9	M	54	8/19.4	13/1	20.5	11
10	F	75	8/19.4	13/1	19	6
11	M	55	10/20	14.5/2.5	19	6
12	F	57	8/19.4	14.5/2.5	19	6
13	F	56	7/19.1	13/1	17.5	6
14	F	60	10/20	13/1	19	6
15	F	64	9/19.7	14.5/2.5	20.5	6
16	M	56	11/20.3	13/1	20.5	6
17	M	67	8/19.4	17/5	22	6
18	F	78	8/19.4	13/1	19	6
19	F	39	7.5/19.2	13/1	20.5	11
20	F	55	7.5/19.2	13/1	19	9
21	F	50	8/19.4	14.5/2.5	22	11
22	M	48	10/20	14.5/2.5	23.5	14
23	F	65	7.5/19.2	14.5/2.5	22	6
24	M	62	10/20	13/1	23	9
25	F	57	9/19.7	13/1	19	6/8
26	F	64	9/19.7	13/1	19	6
27	M	63	8.5/19.5	14.5/2.5	20.5	9
28	F	73	8/19.4	13/1	17.5	12
29	F	70	9/19.7	14.5/2.5	23.5	14
30	M	44	9/19.7	14.5/2.5	22	8
31	F	55	7/19.1	13/1	19	12/20
32	F	80	10/20	13/1	20.5	6/12/24
33	F	56	9/19.7	13/1	19	15/32
34	F	61	9/19.7	13/1	22	8
35	F	80	8/19.4	13/1	20.5	6
36	M	44	10/20	13/1	23.5	6
37	F	55	7.5/19.2	13/1	19	6
38	F	63	8.5/19.5	13/1	19	9
39-right	M	47	8/19.4	14.5/2.5	20.5	6
40-left	M	47	8/19.4	14.5/2.5	20.5	6
41	M	45	9/19.7	13/1	20.5	6
42	M	52	10/20	17/5	22	6
43	F	53	10/20	13/1	20.5	6
44	F	70	10/20	13/1	20.5	8

Ø stands for diameter, while L denotes length.

**Table 2:** Resorption measurement around the right and left sides of the stem in antero-posterior (AP) or lateral (LL) views.

Panel A						B
		AP (mm)		LL (mm)		Mean (mm)
Case	Month	Left	Right	Left	Right	
1	8	3	4	4	5	4
2	6	0	0	0	3	1
2	12	0	2	0	3	1
3	9	2	2	0	0	1
4	6	5	6	7	6	6
5	10	11	7	4	7	7
6	6	0	0	2	0	1
7	15	1	3	5	0	2
8	7	8	7	7	7	7
9	11	5	6	6	6	6
10	6	5	4	5	4	5
11	6	4	2	2	2	3
12	6	6	9	6	9	8
13	6	9	9	9	8	9
14	6	1	3	1	3	2
15	6	3	3	2	3	3
16	6	1	1	2	2	2
17	6	2	1	2	1	2
18	6	2	6	1	1	3
19	11	0	1	1	1	1
20	9	2	3	5	2	3
21	11	2	5	3	2	3
22	14	5	5	5	2	4
23	6	6	5	5	4	5
24	9	8	8	-	-	8
25	6	7	6	7	6	7
25	8	7	6	7	6	7
26	6	0	0	3	0	1
27	9	0	0	0	0	0
28	12	0	2	3	3	2
29	14	4	4	5	5	5
30	8	1	1	2	1	1
31	12	7	7	8	7	7
31	20	6	7	7	7	7
32	6	2	0	0	2	1
32	12	1	0	2	0	1
32	24	5	1	3	0	2
33	15	3	3	-	-	3
33	32	5	4	4	4	4
34	8	4	4	5	5	5
35	6	5	8	6	5	6
36	6	1	3	2	2	2
37	6	3	0	0	0	1
38	9	5	3	5	3	4
39	6	5	3	4	4	4
40	6	1	1	4	3	2
41	6	0	4	4	3	3
42	6	3	3	3	3	3
43	6	0	3	2	3	2
44	8	0	0	8	0	2

“-” for missing radiographs, AP stands for antero-posterior view and LL for lateral view.

**Table 3:** Pearson correlation coefficients for resorption values observed in both AP and LL views on the left and right sides.

	“r”				*p*-Value			
	AP Left	AP Right	LL Left	LL Right	AP Left	AP Right	LL Left	LL Right
AP left	1.00	0.78	0.65	0.76	<0.001	<0.001	<0.001	<0.001
AP right		1.00	0.70	0.85		<0.001	<0.001	<0.001
LL left			1.00	0.67			<0.001	<0.001
LL right				1.00				<0.001

“r” stands for Pearson correlation value, while AP denotes antero-posterior view and LL denotes lateral view.

## Data Availability

The data presented in this study are available on request from the corresponding author.
